# Humanin Attenuates NMDA-Induced Excitotoxicity by Inhibiting ROS-dependent JNK/p38 MAPK Pathway

**DOI:** 10.3390/ijms19102982

**Published:** 2018-09-29

**Authors:** Xiaorong Yang, Hongmei Zhang, Jinzi Wu, Litian Yin, Liang-Jun Yan, Ce Zhang

**Affiliations:** 1 National Key Disciplines, Key Laboratory for Cellular Physiology of Ministry of Education, Department of Neurobiology, Shanxi Medical University, Taiyuan 030001, China; yinlitian@126.com; 2 Department of Environmental Health, Shanxi Medical University, Taiyuan 030001, China; zhhm_108@163.com; 3 Department of Pharmaceutical Sciences, UNT System College of Pharmacy, University of North Texas Health Science Center, Fort Worth, TX 76107, USA; Jinzi.Wu@unthsc.edu (J.W.); Liang-jun.yan@unthsc.edu (L.-J.Y.)

**Keywords:** HN, neuroprotection, NMDA, excitotoxicity, MAPKs, ROS

## Abstract

Humanin (HN) is a novel 24-amino acid peptide that protects neurons against N-methyl-d-aspartate (NMDA)-induced toxicity. However, the contribution of the different mitogen-activated protein kinases (MAPKs) signals to HN neuroprotection against NMDA neurotoxicity remains unclear. The present study was therefore aimed to investigate neuroprotective mechanisms of HN. We analyzed intracellular Ca^2+^ levels, reactive oxygen species (ROS) production, and the MAPKs signal transduction cascade using an in vitro NMDA-mediated excitotoxicity of cortical neurons model. Results showed that: (1) HN attenuated NMDA-induced neuronal insults by increasing cell viability, decreasing lactate dehydrogenase (LDH) release, and increasing cell survival; (2) HN reversed NMDA-induced increase in intracellular calcium; (3) pretreatment by HN or 1,2-bis(2-aminophenoxy)ethane-*N,N,N’,N’*-tetraacetic acid (BAPTA-AM), an intracellular calcium chelator, decreased ROS generation after NMDA exposure; (4) administration of HN or *N*-Acetyl-l-cysteine (NAC), a ROS scavenger, inhibited NMDA-induced JNK and p38 MAPK activation. These results indicated that HN reduced intracellular elevation of Ca^2+^ levels, which, in turn, inhibited ROS generation and subsequent JNK and p38 MAPK activation that are involved in promoting cell survival in NMDA-induced excitotoxicity. Therefore, the present study suggests that inhibition of ROS-dependent JNK/p38 MAPK signaling pathway serves an effective strategy for HN neuroprotection against certain neurological diseases.

## 1. Introduction

Glutamate is a primary excitatory amino acid neurotransmitter in mammalian central nervous system (CNS) and activation of glutamate receptors including N-methyl-d-aspartate (NMDA) receptor plays crucial roles in neural development, synaptic plasticity, transmission, learning and memory [1,2]. Under pathological conditions, the excessive release and accumulation of glutamate over-activates NMDA receptor which cause intracellular Ca^2+^ overload and an enzymatic cascade of events resulting ultimately in cell death through collapse of the mitochondrial membrane, ER stress, increased formation of reactive oxygen species (ROS), and reactive nitrogen species, nitric oxide, lipid peroxidation, and DNA damage [3,4]. This process is commonly known as excitotoxicity [2,5,6]. A wide range of acute and chronic brain injury diseases, such as stroke/ischemia, epilepsy, and certain neurodegenerative disorders have been linked to NMDA receptor-mediated excitotoxicity [7]. Therefore, NMDA-induced excitotoxicity is a useful tool to evaluate neurotoxicity in isolated cells and is a good model of nerve injury that mimics closely the situation in vivo [8].

Mitogen-activated protein kinases (MAPKs), which include extracellular signal-regulated kinase (ERK1/2), c-Jun N-terminal kinase (JNK), and p38 MAPK are evolutionarily conserved enzymes that link cell-surface receptors to intracellular regulatory targets, and have been shown to be involved in the glutamate/NMDA-induced excitotoxicity [9,10,11,12,13]. While the exact mechanism of excitotoxicity is not clear yet, it may however be mediated through activation of ERK1/2, JNK, and p38 MAPK [14,15,16]. Consistent with these findings, we also found the involvement of JNK and p38 MAPK pathways in NMDA-induced cell death of rat cortical neurons [17].

Humanin (HN) is a polypeptide of 24 amino acids that was first isolated from brains of patients with Alzheimer’s disease (AD) [18,19]. HN is best known for its ability to suppress neuronal cell death induced by AD-related insults such as familial AD proteins and neurotoxic Aβ peptides [20]. Recently, evidence has revealed that HN has a broader spectrum of neuroprotective activity [21,22]. For example, HN has been shown to rescue neurons from NMDA-induced toxicity [23,24]. However, it remains sketchy that the neuroprotection of HN is mediated by the MAPK signals. The present study was carried out to investigate the molecular mechanisms underlying the neuroprotection of HN against NMDA neurotoxicity.

## 2. Results

### 2.1. Humanin (HN) Prevents from NMDA-Induced Neuronal Insults

**Figure 1:**
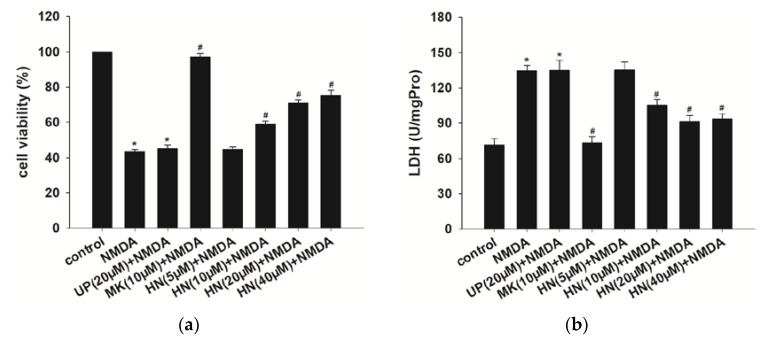

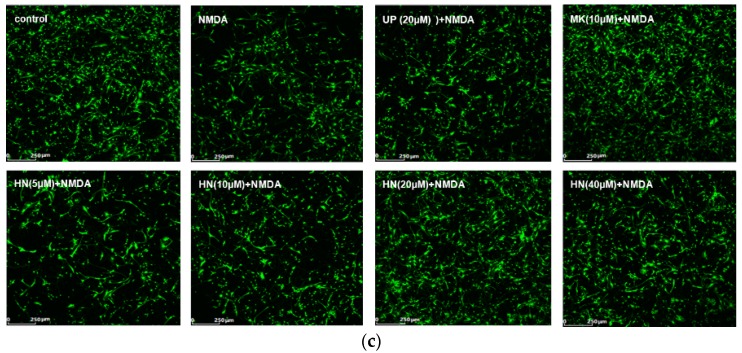
Effects of HN on NMDA-induced neuronal insults. (**a**) Pretreatment of HN (10 μM, 20 μM, 40 μM) improved cell viability compared with NMDA treatment group (*p* < 0.05); (**b**)Pretreatment of HN (10, 20, 40 μM) reduced LDH activity (U/mgPro) in cell supernatant induced by NMDA (*p* < 0.05); (**c**) Pretreatment of HN (10, 20, 40 μM) reversed NMDA-mediated the decrease in cell survival rate (*p* < 0.05). UP: unrelated peptide; MK: MK-801. Each value represents the mean ± S.E.M. of six independent experiments. * *p* < 0.05 vs. control group, # *p* < 0.05 vs. NMDA group.

We found that the optimal excitotoxicity was induced at 24 h after NMDA (100 μM) exposure for two h in primary cortical neurons. As shown in Figure 1, pretreatment with different concentrations (10, 20 and 40 μM) of HN reversed NMDA-induced excitotoxicity including a decrease in cell viability (*p* < 0.05) (Figure 1a), an increase in LDH release (*p* < 0.05) (Figure 1b) and a decrease in cell survival rate (*p* < 0.05) (Figure 1c), as measured by WST-8 assay, LDH assay, and Calcein-AM assay. However, five μM HN had no neuroprotective effects against NMDA-induced neuronal insults (*p* > 0.05). Additionally, pretreatment of 20 μM unrelated peptide (UP), as a control for HN, did not affect the excitotoxic effects (*p* > 0.05). MK-801 (10 μM), a noncompetitive NMDA receptor antagonist, fully abolished the insults trigged by NMDA (*p* < 0.05), supporting that the toxic effects were induced by NMDA. These results suggest that HN counteracts NMDA-induced neuronal insults. In the following experiments, HN was administered at the concentration of 20 μM based on the significant protective effects observed in the 20 μM HN group.

### 2.2. Humanin (HN) Attenuated NMDA-Induced Elevation of Intracellular Calcium

**Figure 2:**
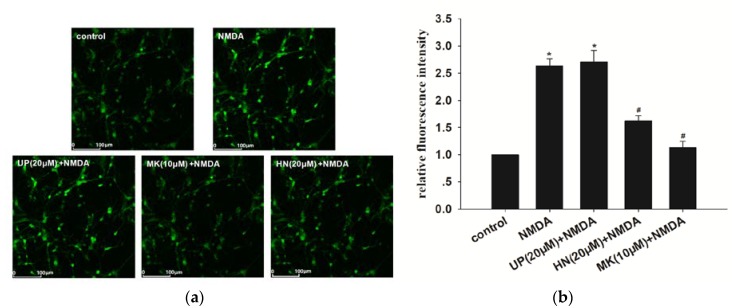
HN suppressed intracellular calcium overload in NMDA-induced excitotoxicity. (**a**) Intracellular Ca^2+^ was measured via live cell imaging; (**b**) Represents bar diagram of relative fluorescence intensity. UP: unrelated peptide; MK: MK-801. Each value represents the mean ± S.E.M. of three independent experiments. * *p* < 0.05 vs. control group, # *p* < 0.05 vs. NMDA group.

As shown in Figure 2, intracellular Ca^2+^ concentration ([Ca^2+^]_i_) after NMDA exposure of cortical neurons is 2.5 fold higher, when compared to the control (*p* < 0.05). MK-801 (10 μM) completely inhibited the increase, suggesting that the elevation of [Ca^2+^]_i_ was caused by NMDA. Pretreatment with HN (20 μM) significantly extenuated intracellular calcium overload triggered by NMDA (*p* < 0.05). We also found that administration of UP (20 μM) did not inhibit NMDA-induced elevation of intracellular calcium (*p* > 0.05).

### 2.3. Decrease in Reactive Oxygen Species Levels by HN, BAPTA-AM or NAC after NMDA Exposure

**Figure 3:**
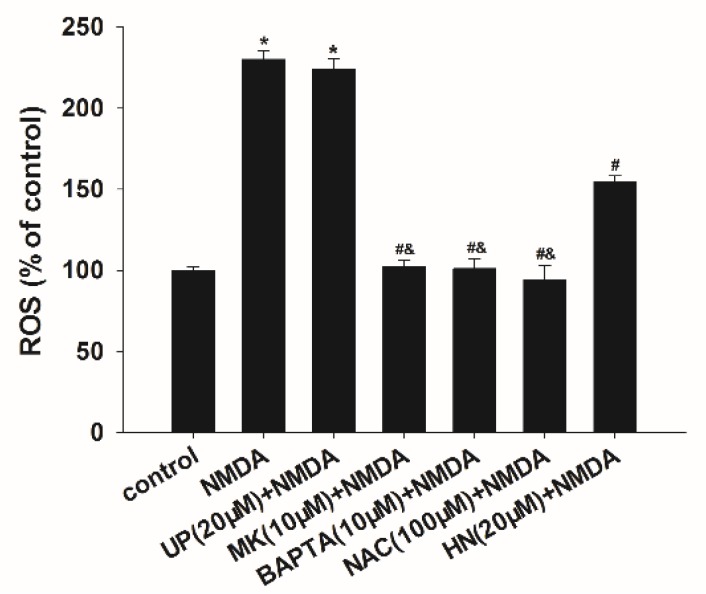
Inhibition of NMDA-induced ROS generation by HN, BAPTA-AM or NAC. UP: unrelated peptide; MK: MK-801; BAPTA: BAPTA-AM; NAC: *N*-Acetyl-l-cysteine. Each value represents the mean ± S.E.M. of three independent experiments. * *p* < 0.05 vs. control group, # *p* < 0.05 vs. NMDA group, & *p* < 0.05 vs. HN + NMDA group.

To clarify the time course of ROS generation during excitotoxicity, we measured the production of ROS and found that NMDA induced an increase of ROS with a peak response at six h (data not shown). NAC (*N*-acetyl-l-cysteine) is commonly used to identify and test ROS (reactive oxygen species) inducers, and to inhibit ROS generation. We respectively examined the effects of ROS scavenger NAC, neuroprotective peptide HN, and intracellular calcium chelator 1,2-bis(2-aminophenoxy) ethane-*N,N,N’,N’*-tetraacetic acid (BAPTA-AM) on ROS generation after NMDA treatment. As shown in Figure 3, administration of HN (20 μM) reduced NMDA-induced production of ROS by 45.71% (*p* < 0.05). MK-801 (10 μM), BAPTA-AM (10 μM) or NAC (100 μM) fully blocked NMDA-induced ROS generation (*p* < 0.05). Pretreatment of UP (20 μM) had no effects on NMDA-induced ROS generation (*p* > 0.05).

### 2.4. NMDA-Induced MAPKs Activation were Attenuated by HN or NAC

**Figure 4:**
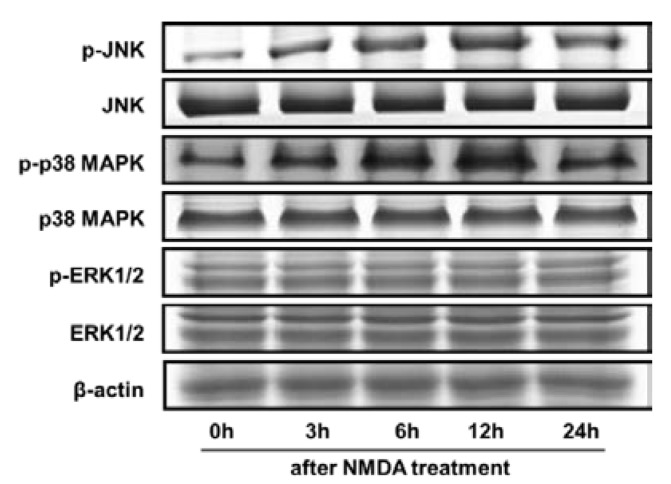
The time course of MAPKs activation in response to NMDA. Representative immunoblots depicting the phosphorylation of JNK, p38 MAPK, and ERK1/2 at 0–24 h after NMDA treatment. However, no significant changes were found in total forms. β-actin was used as a loading control.

To further evaluate the involvement of MAPKs in the excitotoxicity, the phosphorylation of MAPKs was examined at various periods of time after NMDA administration (zero h to 24 h). As shown in Figure 4, phosphorylation levels of JNK and p38 MAPK followed by NMDA were significantly higher at 3 , 6, 12 h, and 24 h than that in the untreated control group (zero h), with a maximal activation at 12 h after NMDA treatment (*p* < 0.05). Total levels of JNK, p38 MAPK and β-actin were virtually unchanged under all of these experimental conditions. The results indicated that NMDA induced the activation of JNK and p38 MAPK in a time-dependent manner. While NMDA did not affect the phosphorylation of ERK1/2 (*p* > 0.05), indicating that ERK1/2 was not involved in the neurotoxicity.

**Figure 5:**
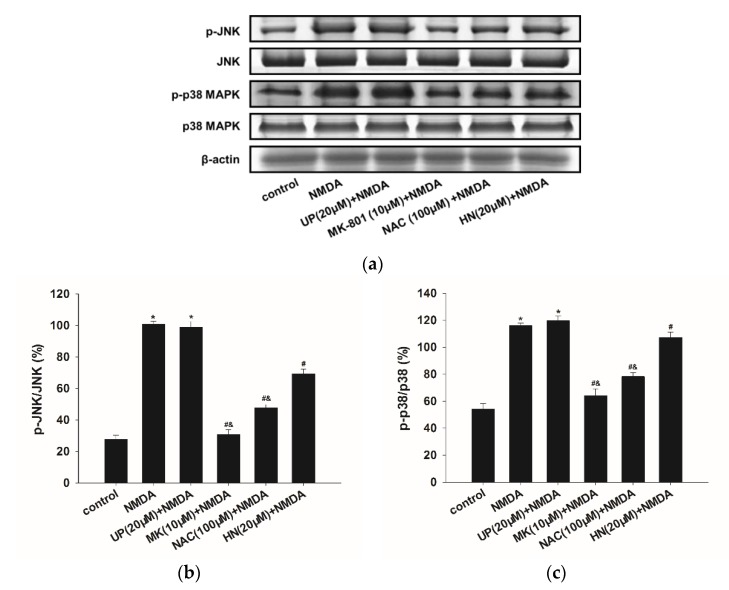
Effects of HN or NAC on the expression levels of phosphorylated JNK and p38 MAPK family proteins in NMDA-induced toxicity. (**a**) Representative Immunoblots and (**b**) densitometry histograms depicting NMDA induced upregulation of p-JNK, and (**c**) p-p38 MAPK proteins and their attenuation by HN or NAC. However, no significant changes were found in total forms. β-actin was used as a loading control. UP: unrelated peptide; MK: MK-801; NAC: *N*-Acetyl-l-cysteine. Each value represents the mean ± S.E.M. of three independent experiments. * *p* < 0.05 vs. control group, # *p* < 0.05 vs. NMDA group, and & *p* < 0.05 vs. HN + NMDA group.

As shown in Figure 5, pretreatment of NAC (100 μM) significantly reduced NMDA-induced JNK and p38 MAPK phosphorylation (*p* < 0.05), which indicated the involvement of ROS in MAPKs signaling pathways. Similarly, HN (20 μM) also suppressed JNK and p38 MAPK phosphorylation (*p* < 0.05). In addition, there was a difference between the NAC + NMDA and HN group (*p* < 0.05). MK-801 (10 μM) completely blocked the effects caused by NMDA (*p* < 0.05). UP (20 μM) did not affect the expression of these proteins when compared with NMDA group (*p* > 0.05).

## 3. Discussion

Results of the present study revealed that HN ameliorated the NMDA-induced death of primary cortical neuron. HN rescued cells from NMDA-induced excitotoxicity by abating intracellular calcium levels, attenuating excessive generation of ROS, and invoking inhibition of MAPK family proteins.

As a well-known neuroprotective factor, HN was first found in certain regions of human AD brain. As has been reported, HN protected neurons against Aβ, okadaic acid-induced neurotoxicity, as well as mutational APP, PS1, and PS2-induced neuronal death [18,25,26] in vitro, and also exerts neuroprotection in the model of AD and myocardial ischemia–reperfusion (I/R) injury in vivo [27,28]. In the present study, we found that preincubation of cortical neurons with HN prevented NMDA-induced excitotoxicity including the decrease in cell viability, the increase in LDH release and cell death triggered by NMDA. These results further confirmed that HN has neuroprotection in NMDA-induced excitotoxicity. Thus, a growing body of evidence has indicated the neuroprotective effects of HN.

We found pretreatment with HN attenuated Ca^2+^ increase trigged by NMDA. It is well known that NMDA contributes to significant increase in intracellular Ca^2+^ concentrations, which induce various pathologic processes such as oxidant stress, ATP depletion, loss of mitochondrial membrane potential that ultimately leads to cell death [3,4]. The Ca^2+^ influx through voltage-gated Ca^2+^ channel does not invoke cell death whereas Ca^2+^ entry via NMDA receptor is linked with Ca^2+^-dependent excitotoxicity [29]. These suggest that the mechanism of HN neuroprotection in rat cortical neuron maybe through antagonism of the NMDA receptor. Nonetheless, there is also a conflicting report showing that HN failed to inhibit intracellular Ca^2+^ overload triggered by NMDA [23,30]. The reason for the discrepancy is unclear, but one possibility could be due to different concentrations of HN used between these two studies. It has been reported that HN exerts protective effects via its putative receptor on the cell membrane, including G protein-coupled receptors, formyl peptide-receptor-like (FPRL)-1 and 2 [31,32]; and a trimeric receptor complex composed of ciliary neurotrophic factor receptor α (CNTFR), gp130, and the IL-27 receptor subunit, WSX-1.7 [33]; and the gp130 receptor [24]. These findings suggest that different mechanisms underlie the HN neuroprotection in different cells in vitro.

In the present study, pretreatment with HN significantly attenuated ROS production in the excitotoxicity. The result agrees with a recent report of an appreciable ability of HN to decrease the ROS levels in NMDA-induced excitotoxicity [23]. Additionally, we also found that administration of BAPTA-AM fully abolished NMDA-induced ROS generation, suggesting that ROS generation triggered by NMDA is Ca^2+^-dependent. It has been shown that overload of intracellular calcium levels induced by excitotoxicity leads to ROS generation, oxidative stress, and ultimately cell death [34]. The present results indicated that HN attenuated intracellular Ca^2+^ influx that in turn decreased ROS levels and then promote cell survival.

We then observed that the phosphorylation levels of JNK and p38 MAPK were significantly up-regulated in a time-dependent manner and reached a maximal response 12 h after NMDA treatment. Furthermore, pretreatment with HN resulted in attenuation of these increased MAPKs levels. The result suggests that HN reverses NMDA-induced neuronal insults through inhibition of JNK and p38 MAPK signaling pathway. Similarly, it has been reported that JNK1 inhibition protected cortical neurons against toxicity and reduced ischemic neuronal insults in rats [35,36,37]. Pharmacological blockade of p38 MAPK attenuated neuronal damage due to cerebral ischemia [38]. Recently, Dar et al. reported that glutamate exposure caused a significant increase in phosphorylation levels of JNK and p38 MAPK in Neuro2a cells and inhibition of these MAPKs proteins had protective effects [16]. Therefore, inhibition of the JNK and p38 MAPK pathways protect against excitotoxic neuronal death in vitro and stimulating the JNK and p38 MAPK pathways aggravate neuronal death. Nonetheless, we observed that NMDA did not affect ERK1/2 phosphorylation. The contribution of ERK1/2 pathway to neuronal insults in vivo and in vitro models are still so controversial. For example, ERK1/2 activation participated in neuronal insults and inhibition of ERK afforded neuroprotection against excitotoxicity and cerebral ischemia [39,40], while it is also reported that facilitation of ERK activation prevents glutamate-induced apoptosis in SCN2.2 cells [41]. The role of ERK on cell survival/death seems to be associated with the model system and injury paradigm [42].

Further observation showed that pretreatment of NAC fully prevented ROS production and markedly ameliorated NMDA-induced JNK and p38 MAPK activation. Therefore, it suggests that ROS generation is required for MAPKs activities in the excitotoxicity model. ROS has been implicated in the promotion of cell death through JNK signaling pathway due to various oxidative stress exposure and effects of JNK and p38 MAPK depend on elevated ROS production [43,44,45]. Likewise, studies have shown that ROS production induced by Palmitate (PA) contributed to the activation of JNK and p38 MAPK, which results in autophagy and apoptosis of H9c2 cells [46]. Based on the above observation that HN decreased ROS levels after NMDA exposure, it is supposed that HN’s neuroprotective strategies against NMDA-induced excitotoxicity in cortical neuron are capable of inhibiting ROS-dependent JNK and p38 MAPK activation.

Taken together, our study indicates that over-activation of NMDA receptor may trigger high Ca^2+^ influx and subsequent ROS generation, leading to JNK and p38 MAPK activation, resulting, ultimately, in an enzymatic cascade of events and cell death, and pretreatment of HN attenuates NMDA-induced excitotoxicity by inhibiting Ca^2+^-ROS-JNK/p38 MAPK signaling pathway. We cannot, however, rule out the possibility that other pathways are also involved in NMDA toxicity and HN-mediated neuroprotection. Nonetheless, this study further elucidates the mechanism underlying the HN-mediated neuroprotective signal. Understanding the mechanism for the rescue function of HN would provide an insight into the novel avenue toward the development of neurodegenerative disease therapies.

## 4. Materials and Methods

### 4.1. Reagents

Synthetic Humanin (HN) and unrelated peptide (UP) were purchased from Sangon biotech (Shanghai China). The sequences of HN and UP are MAPRGFSCLLLLTSEIDLPVKRRA and IYMCILTVYPAEAISQWGRDLAVD, respectively. The Neurobasal/B27 and DMEM/F-12 were purchased from Gibco-BRL (Grand Island, NY, USA). Poly-D-lysine (MW 150,000–300,000), trypsin, arabinoside cytosine, Calcein-AM, *N*-Acetyl-l-cysteine (NAC), NMDA, and MK-801 were all purchased from Sigma-Aldrich (St. Louis, MO, USA). Cell Counting Kit-8 (CCK-8) was from Dojindo (Kumamoto, Japan), and Kit of LDH was from Njjcbio (Nanjing, China). Fluo-3AM and 2’,7’-dichlorodihydrofluorescein diacetate (H2DCFDA) were from Invitrogen (Carlsbad, California, USA). MAPK and Phospho-MAPK Family Antibody Sampler Kits were obtained from Cell Signaling Technology (Beverly, MA, USA). β-actin was from Santa Cruz Biotechnology (Santa Cruz, CA, USA). New born Wistar rats were from the Experimental Animal Center of Shanxi Medical University.

### 4.2. Animal and Cell Culture

1–3-day-old Wistar rats were supplied by the Research Animal Center of Shanxi Medical University, with approval of the Shanxi Committee on Ethics of Animal Research (approval No. SYXK[JIN]2015-0001, approved on 4 March 2015). Primary cortical cells were isolated from Wistar rats and were cultured as previously described [47]. In brief, cortical neurons from rats anesthetized with ketamine (intraperitoneal injection, 100 mg/kg, 3 min) were dissected and digested in 0.025% trypsin, followed by centrifugation at 800× *g* for 5 min. Cells were re-suspended in Neurobasal/B27 medium, and cultured at 37 °C in 5% CO_2_. Arabinoside cytosin (10 μM) was added after 24 h to inhibit non-neuronal cell growth. Only mature neuronal cells (10–12 days) in vitro were used for the experiments.

### 4.3. Treatments

After overnight incubation allowing the cells to reach 80% confluency, cultured cortical neurons were treated with 100 μM NMDA for 2 h. HN (20 μM) or NAC (*N*-Acetyl-l-cysteine) (100 μM) was added to cultures 16 h prior to NMDA induction. BAPTA-AM (10 μM) was respectively added 1 h before NMDA treatment. MK-801 (10 μM) and NMDA were simultaneously added to Mg^2+^-free Locke’s buffer in the NMDA + MK-801 group. Control cells were incubated with drug-free Mg^2+^-free Locke’s buffer and grown at 37 °C in an atmosphere containing 5% CO_2_.

### 4.4. Cell Viability Assay

Cells were seeded in 96-well plates, and cell viability was assayed 24 h after NMDA exposure. Ten microliters of cck-8 solution was administrated into each well, following incubation for 2 h at 37 ˚C. Absorbance at 490 nm was measured using a microplate reader (Packard Instrument Company, Meriden, CT, USA).

### 4.5. Lactate Dehydrogenase (LDH) Assay

Cell injury induced by NMDA was quantitatively assessed by the measurement of LDH released from damaged cells. In this study, we used serum-free medium when detected LDH [48,49]. The formula was Neurobasal 96.75 mL, B27 2 mL, l-glutamine 0.25 mL, and mycillin 1 mL to configure 100 mL serum-free medium. This combination had been shown to reduce glia to less than 0.5 %. The cells were plated in 24-well plates. At 24 h after NMDA exposure, 500 μL of supernatant was collected from each well and mixed with 1.3 mL of NADH (0.217 mmoL/L) and 1.3 mL of sodium pyruvate (1.77 mmoL/L) in the modiWed Krebs–Henseleit buffer for 30 s at 37 °C. The absorbance was measured at 450 nm using a microplate reader and the LDH activity was expressed as U/mgPro.

### 4.6. Calcein-AM Assay

Calcein-AM solution (20 μM) was added to coverslips, the cells were incubated at 37 °C for 30 min and were washed twice with PBS. Then, the cells were examined under fluorescence microscopy (IX51, Olympus, Tokyo, Japan).

### 4.7. Confocal Calcium Imaging/Measurement

Intracellular free Ca^2+^ was measured with fluorescent dye Fluo-3/AM utilizing confocal laser scanning microscopy (TCS SP5, Leica, Mannheim, Germany). Briefly, cells were incubated with 3 μM Fluo-3/AM for 30 min at 37 °C in the dark. The cells were washed with PBS to remove any unbound dye. Live video images of selected neurons under microscopic field (approximately 10–30 neurons per field) were recorded with a confocal laser-scanning microscope. Excitation wavelength of 490 nm and emission wavelength of 528 nm were selected for the fluo-3 fluorescence. Quantitative analysis of the images was performed by Leica image analysis software by calculating the florescence intensity of the region of interest (ROI).

### 4.8. Measurement of Reactive Oxygen Species (ROS)

Intracellular ROS level was measured using the fluorescent probe H2DCFDA. Briefly, the cells (1 × 10^5^) in 6-well plates were incubated with 10 μM H2DCFDA for 30 min at 37 °C in the dark. After incubation, the cells were washed with 1× PBS, and resuspended in PBS, and analyzed by flow cytometry. The fluorescence emitted at 488 nm was measured with a FACS Calibur flow cytometer (BD FACSAriaT) and analyzed using the CELL Quest software. Ten thousand cells were examined for each sample. The values were expressed as percentage of fluorescence in the control. ROS levels were expressed as a percentage of control.

### 4.9. Western Blot Analysis

Cells were lysed in a lysis buffer [10 mM Tris–HCl (pH 7.4), 1 mM EDTA, and 1% Triton X-100]. Cleared cell lysates were obtained after centrifugation at 10,000× *g* for 30 min at 4 °C. After measurement of protein concentration using a BCA Protein Assay kit, cell lysates (30–50 μg/lane) were subjected to SDS-PAGE, and separated proteins were electrotransferred to nitrocellulose membranes. The membranes were washed in Tris-buffered saline (TBS) containing 0.1% Tween 20 and 3% bovine serum albumin (BSA). The membranes were incubated overnight at 4 °C in TBS containing 3% BSA and one of the following primary antibodies: p-ERK1/2, ERK1/2, p-JNK, JNK, p-p38 MAPK, p38 MAPK, and β-actin. Subsequently, the labeled proteins were incubated with an HRP-conjugated goat anti-rabbit IgG for 2 h. Blots were developed with the ECL chemiluminescence system and were captured on autoradiographic films (Kodak Image Station 440, Kodak GmbH, Stuttgart, Germany). Films were scanned and densitometric analysis of the bands was performed with AlphaEase Image Analysis Software (Version 5.0.1, Alpha Innotech, San Leandro, CA, USA).

### 4.10. Statistical Analysis

Statistical analyses were performed using SPSS 21.0 (SPSS Inc., Chicago, IL, USA). The data were expressed as means ± S.E.M. of at least three independent experiments. One-way analysis of variance (ANOVA) with bonferroni post-hoc test was used for statistical comparisons. *p* < 0.05 was considered to be significant.

## Abbreviations

CNS	central nervous system
HN	Humanin
UP	unrelated peptide
NMDA	N-methyl-d-aspartate
MK-801	5S,10R)-(+)-5-Methyl-10,11-dihydro-5H-dibenzo[a,d]cyclohepten-5,10-imine maleate
MAPKs	Mitogen- activated protein kinases
ERK1/2	extracellar signal-regulated protein kinase
JNK	c-Jun N-terminal kinase
BAPTA-AM	1,2-bis(2-aminophenoxy)ethane-*N,N,N’,N’*-tetraacetic acid
LDH	lactate dehydrogenase
AD	Alzheimer’s disease
ROS	reactive oxygen species
NAC	*N*-Acetyl-l-cysteine
H2DCFDA	2’,7’-dichlorodihydrofluorescein diacetate
FPRL	formyl peptide-receptor-like
CNTFR	ciliary neurotrophic factor receptor α
